# The Composition of Fatty Acids in Ostrich Meat Influenced by the Type of Packaging and Refrigerated Storage

**DOI:** 10.3390/molecules24224128

**Published:** 2019-11-15

**Authors:** Olaf K. Horbańczuk, Małgorzata Moczkowska, Joanna Marchewka, Atanas G. Atanasov, Marcin A. Kurek

**Affiliations:** 1 Division of Engineering in Nutrition, Department of Technique and Food Product Development, Warsaw University of Life Sciences (WULS-SGGW) 159c Nowoursynowska, 02-776 Warsaw, Poland; malgorzata_moczkowska@sggw.pl (M.M.); marcin_kurek@sggw.pl (M.A.K.); 2 Institute of Genetics and Animal Breeding of the Polish Academy of Sciences, 05-552 Jastrzebiec, Poland; j.marchewka@ighz.pl (J.M.); atanas.atanasov@univie.ac.at (A.G.A.); 3 Institute of Neurobiology, Bulgarian Academy of Sciences, 23 Acad. G. Bonchev str., 1113 Sofia, Bulgaria; 4 Department of Pharmacognosy, University of Vienna, 1090 Vienna, Austria

**Keywords:** ostrich meat, PUFA, vacuum, modified atmosphere packaging, storage time

## Abstract

Ostrich meat is a high-quality dietetic product, however, it is very sensitive to deterioration during storage. The aim of this study was to assess the effect of packaging systems on the fatty acid (FA) profiles in ostrich meat during refrigerated storage. The systems were: Vacuum packaging (VP) and modified atmosphere packaging (MAP) in two combinations of gases: MAP1 (40% O_2_/40% CO_2_/20% N_2_) and MAP2 (60% O_2_/30% CO_2_/10% N_2_). Samples were taken from the *M. ilifibularis* (IF) muscles of eight ostriches in each treatment group. The packs were stored in a refrigerator at 2 °C and analyzed at 0, 4, 8, 12 and 16 days. The packaging conditions and storage time had an impact on the concentration of bioactive compounds such as polyunsaturated fatty acids (PUFA), including n-3 such as C18:3, C20:5 (EPA) and C22:6 (DHA). The least changes in composition of n-3 and the sum of PUFA were recorded in ostrich meat packaged in vacuum, followed by that packaged using MAP1 and MAP2. The sum of n-6 PUFAs decreased significantly by 2.1% for MAP2, and only by 0.7% for vacuum packaging as the experiment progressed. A significant deterioration of these compounds was observed in all package systems, especially from day 12 until day 16 of storage.

## 1. Introduction

A growing demand for ostrich meat has been observed globally over the last decades [1,2,3,4,5]. Popularity of ostrich meat can be attributed to the fact that it is recognized as a dietetic product, characterized by low fat levels (less than 2 mg/100 g) [6], a lower calorific value of 390 kJ/100 g as compared to beef (517 kJ/100 g), as well as a high share of polyunsaturated fatty acids (PUFA) [4]. For example, the PUFA content in ostrich meat (24–38% sum of fatty acids in total) reached higher levels compared to chicken meat −19% and beef −4.8% (sum of fatty acids in total) [7]. In addition, the ratio of saturated fatty acids (SFA) to monounsaturated fatty acids (MUFA) to PUFA in ostrich meat is often 1:1:1, which is very important from a dietetic point of view. Cholesterol content in ostrich meat is also lower in comparison to beef and chicken meat, with 54 mg/100 g as reported for raw ostrich meat [4]. Moreover, ostrich meat is high in iron (4 mg/100 g), selenium, vitamin B, and biologically active peptides, such as anserine [8,9,10]. Therefore, this type of meat has gained the appreciation of consumers that are aware of its high dietary quality. However, the abundance of nutrient compounds in ostrich meat, including bioactive molecules such as PUFAs makes this meat highly susceptible to oxidation processes [11,12,13,14,15,16,17]. The oxidative deterioration of PUFAs causes the formation of hydroperoxides, which leads to formation of aldehydes, ketones, and other oxygenated compounds, which are then considered to be responsible for the decrease of meat quality during storage. Lipid oxidation is an important factor related to shelf life and consumer acceptance of fresh meat [7]. One of the possible measures of retaining the optimal ostrich meat composition may include the use of an effective packaging type to protect its nutritional properties [16]. However, up to date research on ostrich meat shelf life quality, packaging type and its behavior during storage in relation to its bioactive molecule composition, especially PUFAs, is still lacking. Therefore, the aim of this study was to evaluate the influence of packaging systems: Vacuum packaging (VP) and modified atmosphere packaging (MAP) in two combinations of gases: MAP1 (40% O_2_/40% CO_2_/20% N_2_) and MAP2 (60% O_2_/30% CO_2_/10% N_2_), on the fatty acid profiles of ostrich meat during refrigerated storage of up to 16 days.

## 2. Results and Discussion

### 2.1. Chemical Composition

The chemical composition of ostrich meat as an initial characterization of samples based on NIR spectroscopy is presented in Table 1. The data are in agreement with Hoffman et al. [6], who found ostrich meat contained 21.65%, 1.95%, and 1.2% of protein, fat and ash, respectively. Similar values in the proximate composition of ostrich meat were also reported by Majewska et al. [18]. 

### 2.2. Fatty Acid Profile

As shown in Table 2, the concentration of individual SFA in ostrich meat did not differ significantly depending on the storage duration and type of packaging (except for C16:0). The significant changes in C16:0 levels between day 0 and 16 were only observed when meat was packaged under MAP2 conditions, while for vacuum packaging the change was by only 0.2%, followed by a 0.4–1% change in MAP (Table 2). The same effects were observed for MUFA (Table 3). These fatty acids were not generally affected by the type of packaging and storage duration. Only the palmitoleic acid (C16:1) and eicosenoic acid (C20:1 n9) were influenced by the packaging conditions and storage duration, and decreased over the course of storage time for MAP2 (C16:1 decreased from 7.90 percent on the initial day to 7.00 percent on day 16, while the decrease for MAP1 and vacuum system was seen to a lesser extent). Similar tendencies regarding palmitoleic acid were reported by Conchillo et al. [17] in chicken meat during 6 days of storage time at the temperature of 4 °C. The results of our investigation are also in agreement with the study carried out on ostrich meat packaged in vacuum and skin pack, and stored up to 14 days by Polawska et al. [19], in which there were no significant differences in MUFA values between the two systems of packaging: Vacuum vs skin pack.

Ostrich meat is a relatively rich source of valuable PUFAs including n-3, as compared to beef and chicken [20,21,22], which could be advantageous in its positive marketing. Intake of n-3 fatty acids reduces the incidence of coronary disease, and allows for greater antithrombotic and antiatherogenic effects than does the intake of the corresponding n-6 polyunsaturated fatty acids [4,23,24,25]. In the current study, the packaging conditions had a significant impact on the concentration of n-3 polyunsaturated fatty acids (Table 4), especially on C18:3, C20:4, C20:5 (EPA - eicosapentaenoic acid), C22:6 (DHA - docosahexaenoic acid) which were influenced by the type of packaging and refrigerator storage duration. In the case of C18:3, the significant change was observed for MAP2 (Table 4). Up to day 8 of the experiment, the changes were not statistically significant, however, a decrease in fatty acid (FA) content resulting from the process of oxidation began to be noted between days 8, 12 and 16 of the experiment. It was noticed that vacuum packaging maintained the levels of DHA in ostrich meat without any changes over the course of the experiment. In the case of EPA, the decrease of its concentration over the 16 days of the experiment was also not statistically significant. On the other hand, MAP1 allowed the above mentioned FA levels to be maintained up to day 12, with a drop to 0.43% in EPA afterwards. When packaged using MAP2, the level of EPA decreased significantly from the initial 0.57% down to 0.33% on day 16. In line with this, a decrease in EPA during refrigerated storage was also shown in the study on chicken meat conducted by Conchillo et al. [17]. 

Similar tendencies were reported for the sum of PUFA, including total n-3 PUFA (Table 5). The sum of n-6 PUFAs in our study decreased over the course of the experiment by 2.14% for MAP2, and only by 0.7% for vacuum packaging. This further influenced the n-6/n-3 ratio, which in the case of vacuum packaging did not change, but increased significantly for MAP2 (Table 5). A tendency for a decrease in the content of PUFA during storage was shown by Poławska et al. [18], who assessed the fatty acid profile of ostrich meat enriched in n-3 fatty acids, packaged in different types of packaging and stored in a refrigerator for 14 days. In the investigation conducted on beef meat by Mahecha et al. [26], a decrease in PUFA content of the longissimus muscle of German Simmental was demonstrated after 14 days of storage. A decline in the PUFA content during storage was also reported by Echarte et al. [27] in pork meat, as well as by Dal Bosco et al. [28] in rabbit meat after 8 days of storage. Interesting results were also obtained in another study done by Dal Bosco et al. [29] who investigated the effect of diet (enriched with linseed) and packaging system on the oxidative status and polyunsaturated fatty acid content of rabbit meat during 10 days of storage under chilled conditions. After linseed supplementation of the rabbit diet, the concentration of PUFA in fresh rabbit meat was significantly higher (36.9 vs 29.5% in total sum of FA) than in control group. However, the higher level of total PUFA’s including C18:3, C20:5 (EPA) and C22:6 (DHA) determined a significantly higher lipid oxidation and the decrease in PUFA’s content in meat during 10 days of storage. Thus, the results of our study are in agreement with the findings of Dal Bosco et al. [29]. On the other hand, the use of stereospecific analysis of FA in the study on rabbit meat done by D’Arco et al. [30] had positive effects on the FA profile, especially on the long chain PUFAs. 

Another study investigating the changes in fatty acid composition of ostrich meat enriched with linseed and rapeseed—packaged in vacuum, frozen (−20 °C) and stored for 120 days [31]—showed a decrease in PUFA levels of the meat, as related to frozen storage duration. The results suggest that although freezing (−20 °C) is an acceptable method for preservation of ostrich meat enriched with n-3 fatty acids, it should only be done for up to 60 days, as the negative changes in PUFA profiles began to be observed only in the second period of storage (61–120 days). 

## 3. Materials and Methods 

### 3.1. Samples and Packaging

Meat samples were taken from the *Musculus ilifibularis* (IF) of ostriches slaughtered at the age of 10–12 months, at a weight range between 90 to 95 kg (8 in each group). The IF muscle was excised (including the removal of external fat and visible connective tissue) from the carcasses 24 h after slaughter and was cut into 2.5 cm thick steaks starting from the proximal side (sample weight: 150 ± 15 g). The steaks were then randomly assigned to one of three packaging conditions:

(a) Vacuum packaging systems: Each steak was packaged individually in polyamide/polyethylene (PA/PE) bags (thickness: 90μm; size: 20/70 mm; oxygen permeability: 50 cm^3^/m^2^/24 h; CO_2_ permeability: 140 cm^3^/m^2^/24 h; water vapor permeability: 6–8 g/m^2^/24 h) within 1 min after cutting, and vacuum packaged using a Vac-20SL2A packaging machine (Edesa Hostelera S.A., Barcelona, Spain). The in-package vacuum level was 2.5 kPa.
(b) Modified atmosphere packaging (MAP) in two combinations of gases, 40% O_2_/40% CO_2_/20% N_2_ (MAP1) and 60% O_2_/30% CO_2_/10% N_2_ (MAP2). The steaks were placed on polyethylene terephthalate/polyethylene (PET/PE) trays (parameters: 187 × 137 × 50 mm), and the film used was a 44 μm thick polyethylene terephthalate/cast polypropylene + antifog (PET/CPP + AF) laminate with maximum oxygen permeability not exceeding 10 cm^3^/m^2^/24 h/bar (EC04, Corenso, Helsinki, Finland). Samples were packaged with an M3 packaging machine (Sealpack, Oldenburg, Germany).

The packs were stored in a refrigerator at 2 °C for the duration of the experiment, for up to 16 days. Samples were collected in three independent replicates, and analyzed at 0 (24 h after slaughter), 4, 8, 12 and 16 days of storage.

### 3.2. Chemical Composition of Samples

NIR spectroscopy analysis was performed using NIRFlex Solids N-500 spectrophotometer (BUCHI Labortechnik GmbH, Switzerland) to examine the chemical composition of the samples. Results were expressed as percentage of protein, fat, moisture and ash. All six scans of each sample were examined for consistency and then averaged.

### 3.3. Fatty Acid Analyses

The fat from the meat (intramuscular fat) was isolated using the Folch [32] method to determine fatty acid composition. Fatty acids were extracted from homogenized samples (5 g) of muscles with the chloroform-methanol (2:1 *v*/*v*). Fatty acid methyl esters (FAME) were analyzed using a GC-7890 AGILENT gas chromatograph equipped with a 60 m HEWLETT – PACKARD-88 capillary column (AGILENT J&W GC Columns, USA- Part Number: 112-8867E) with 0.25 mm inner diameter and 0.20 μm film thickness. A 1 μL sample was injected at a split ratio of 1:40. Helium was used as a carrier gas at a flow rate of 50 mL min^−1^. The injector and detector were both maintained at 260 °C. Column oven temperature was programmed to increase from 140 °C (held for 5 min) at a rate of 4 °C min^−1^ to 190 °C, and then to 215 °C at a rate 0.8 °C min^−1^. Individual fatty acids were identified by comparison of retention times to those of a standard FAME mixture (SUPELCO 37 Component FAME Mix, SIGMA−ALDRICH Co), and expressed as the percentage (%) of FAME. A standard mixture containing all FAs was used to prepare the stock solution. Calibration curves were produced from all working standard sets by diluting half a volume with n-hexane. The stock solution of the internal standard was prepared by dissolving 0.1 g eicosanoic acid (C20:0) in 10 mL hexane. 

### 3.4. Statistical Analysis

A generalized linear mixed model analysis (repeated measures ANOVA) was performed on all measured parameters including selected SFA, MUFA, PUFA and sum of fatty acid (%), and their ratio, in order to determine the effect of packaging treatment as a fixed factor and storage time as a repeated measure, as well as their interaction, on each variable. There were no outliers present in the dataset. Normality and homogeneity of residual variance assumptions were checked and met by all variables under investigation. A generalized linear mixed model analysis (repeated measures ANOVA) was performed on all measured parameters in order to determine the effect of packaging treatment and storage time on each variable. The validity of the models was tested using Akaike’s information criterion. PROC GLIMMIX of SAS v 9.4 (SAS Institute Inc., Cary, NC, USA) including the Tukey adjustment option was used to conduct the analysis. The least square means for all significant effects in the models (*p* ≤ 0.05) were computed using the LSMEANS option. The trend of a significant effect was considered for *p* < 0.10.

## 4. Conclusions

The results of our study demonstrated that the type of packaging system: Vacuum packaging and modified atmosphere packaging in two combinations of gases: 40% O_2_/40% CO_2_/20% N_2_ (MAP1) and 60% O_2_/30%CO_2_/10% N_2_ (MAP2), as well as storage duration, had an impact on the fatty acid concentration, mainly PUFAs, including n-3 such as C18:3, C20:5 (EPA) and C22:6 (DHA). In the above mentioned packaging conditions, significant decreases in FA levels were shown in the last quarter of the storage period (from day 12 to day 16 of storage), while the highest oxidation stability related to the n-3 sums of PUFA was recorded in ostrich meat packaged under vacuum, followed by that packaged using MAP1 and MAP2.

## Figures and Tables

**Table 1:** Chemical composition (g/100 g edible portion) of raw ostrich meat (Mean ± SE).

Parameters	Mean ± SE ^a^
Moisture	75.40 ± 0.26
Fat	1.95 ± 0.03
Protein	21.50 ± 0.11
Ash	1.15 ± 0.01

^a^ Standard error.

**Table 2:** Saturated fatty acids (SFA) composition (%) in the estimated total sum of fatty acid (FA) (mean ± SE) in ostrich meat as related to the type of packaging and refrigerated storage.

Fatty Acid (%)	Method	Day				
		0	4	8	12	16
C14:0	MAP1	0.57 ± 0.12	0.57 ± 0.09	0.57 ± 0.09	0.57 ± 0.09	0.58 ± 0.12
	MAP2	0.57 ± 0.12	0.57 ± 0.09	0.58 ± 0.12	0.59 ± 0.11	0.58 ± 0.12
	Vacuum	0.57 ± 0.12	0.57 ± 0.10	0.57 ± 0.08	0.57 ± 0.06	0.58 ± 0.05
C15:0	MAP1	0.02 ± 0.00	0.02 ± 0.00	0.02 ± 0.00	0.02 ± 0.00	0.02 ± 0.00
	MAP2	0.02 ± 0.00	0.02 ± 0.00	0.02 ± 0.00	0.02 ± 0.00	0.02 ± 0.00
	Vacuum	0.02 ± 0.00	0.02 ± 0.00	0.02 ± 0.00	0.02 ± 0.00	0.02 ± 0.00
C16:0	MAP1	21.37 ± 0.21	21.44 ± 0.10	21.53 ± 0.12	21.59 ± 0.12	21.71 ± 0.14b
	MAP2	21.37 ± 0.21B	21.42B ± 0.10	21.45B ± 0.13	21.52B ± 0.12	22.72 ± 0.07Aa
	Vacuum	21.37 ± 0.21	21.42 ± 0.12	21.47 ± 010	21.50 ± 0.09	21.54 ± 0.15b
C17:0	MAP1	0.12 ± 0.00	0.12 ± 0.00	0.13 ± 0.00	0.12 ± 0.00	0.11 ± 0.00
	MAP2	0.12 ± 0.00	0.11 ± 0.00	0.11 ± 0.00	0.11 ± 0.00	0.11 ± 0.00
	Vacuum	0.12 ± 0.00	0.12 ± 0.00	0.12 ± 0.00	0.11 ± 0.00	0.11 ± 0.00
C18:0	MAP1	9.81 ± 0.08	9.84 ± 0.09	9.87 ± 0.08	9.90 ± 0.06	9.99 ± 0.08
	MAP2	9.81 ± 0.08	9.88 ± 0.01	9.91 ± 0.04	9.95 ± 0.10	10.25 ± 0.11
	Vacuum	9.81 ± 0.08	9.83 ± 0.09	9.85 ± 0.02	9.88 ± 0.13	9.92 ± 0.13

Mean values bearing different letters either for each day within rows (A, B) or between packaging systems within columns (a, b) differ significantly at *p* < 0.05. ND, not detectable = [100 − ∑(SFA + MUFA + PUFA)]. MUFA - monounsaturated fatty acids, PUFA - polyunsaturated fatty acids. MAP1 (40% O2/40% CO2/20% N2) and MAP2 (60% O2/30% CO2/10% N2).

**Table 3:** MUFA composition (%) in the estimated total sum of FA (mean ± SE) in ostrich meat as related to the type of packaging and refrigerated storage.

Fatty Acid (%)	Method	Day				
		0	4	8	12	16
C14:1	MAP1	0.08 ± 0.00	0.09 ± 0.00	0.09 ± 0.00	0.08 ± 0.00	0.06 ± 0.00
	MAP2	0.08 ± 0.00	0.08 ± 0.00	0.07 ± 0.00	0.08 ± 0.00	0.08 ± 0.00
	Vacuum	0.08 ± 0.00	0.08 ± 0.00	0.07 ± 0.00	0.08 ± 0.00	0.06 ± 0.00
C15:1	MAP1	0.17 ± 0.01	0.17 ± 0.00	0.17 ± 0.00	0.16 ± 0.01	0.16 ± 0.01
	MAP2	0.17 ± 0.01	0.17 ± 0.00	0.16 ± 0.01	0.15 ± 0.00	0.15 ± 0.01
	Vacuum	0.17 ± 0.01	0.17 ± 0.01	0.17 ± 0.00	0.16 ± 0.01	0.16 ± 0.01
C16:1	MAP1	7.90 ± 0.09A	7.92 ± 0.07A	7.59 ± 0.09AB	7.33 ± 0.07AB	7.15 ± 0.07Bb
	MAP2	7.90 ± 0.09A	7.87 ± 0.01A	7.70 ± 0.04A	7.41 ± 0.14B	7.06 ± 0.06Bb
	Vacuum	7.90 ± 0.09A	7.93 ± 0.08A	7.65 ± 0.06AB	7.52 ± 0.11B	7.43 ± 0.08Ba
C18:1 n9t	MAP1	0.25 ± 0.01	0.25 ± 0.00	0.26 ± 0.01	0.28 ± 0.01	0.27 ± 0.01
	MAP2	0.25 ± 0.01	0.27 ± 0.01	0.27 ± 0.01	0.28 ± 0.01	0.28 ± 0.01
	Vacuum	0.25 ± 0.01	0.25 ± 0.01	0.26 ± 0.01	0.26 ± 0.00	0.27 ± 0.01
C18:1 n9c	MAP1	29.96 ± 0.15	30.02 ± 0.17	29.75 ± 0.18	29.29 ± 0.14	28.98 ± 0.17
	MAP2	29.96 ± 0.15	30.05 ± 0.22	29.94 ± 0.17	29.72 ± 0.14	29.56 ± 0.13
	Vacuum	29.96 ± 0.15	29.93 ± 0.14	29.75 ± 0.15	29.41 ± 0.10	29.18 ± 0.09
C20:1 n9	MAP1	0.21 ± 0.02B	0.23 ± 0.01B	0.26 ± 0.01AB	0.27 ± 0.01AB	0.30 ± 0.01Aa
	MAP2	0.21 ± 0.02	0.23 ± 0.01	0.26 ± 0.01	0.25 ± 0.02	0.24 ± 0.00b
	Vacuum	0.21 ± 0.02B	0.21 ± 0.00B	0.23 ± 0.00AB	0.25 ± 0.01AB	0.27 ± 0.01Aab

Mean values bearing different letters either for each day within rows (A, B) or between packaging systems within columns (a, b) differ significantly at *p* < 0.05. ND, not detectable = [100 − ∑(SFA + MUFA + PUFA)].

**Table 4:** PUFA composition (%) in the estimated total sum of FA (mean ± SE) in ostrich meat as related to the type of packaging and refrigerated storage.

Fatty Acid (%)	Method	Day				
		0	4	8	12	16
C18:2 n-6c	MAP1	18.70 ± 0.10	18.61 ± 0.12	18.23 ± 0.09	17.97 ± 0.18	17.61± 0.11
	MAP2	18.70 ± 0.10	18.71 ± 0.14	18.04 ± 0.12	17.73 ± 0.20	17.2 ± 0.11
	Vacuum	18.70 ± 0.10	18.67 ± 0.01	18.44 ± 0.07	18.11 ± 0.11	17.92 ± 0.17
C18:3 n-3	MAP1	1.98 ± 0.04	1.97 ± 0.02	1.94 ± 0.04	1.89 ± 0.02a	1.83 ± 0.03a
	MAP2	1.98 ± 0.04A	1.95 ± 0.01A	1.89A ± 0.02A	1.75 ± 0.06Bb	1.46 ± 0.03Cb
	Vacuum	1.98 ± 0.04	1.97 ± 0.03	1.95 ± 0.02	1.93 ± 0.03a	1.94 ± 0.03a
C20:2 n-6	MAP1	0.19 ± 0.01	0.19 ± 0.00	0.17 ± 0.00	0.17 ± 0.00	0.16 ± 0.00
	MAP2	0.19 ± 0.01	0.19 ± 0.01	0.18 ± 0.02	0.17 ± 0.00	0.18 ± 0.00
	Vacuum	0.19 ± 0.01	0.19 ± 0.01	0.19 ± 0.00	0.19 ± 0.01	0.17 ± 0.00
C20:3 n-6	MAP1	0.21 ± 0.01A	0.21 ± 0.00A	0.19 ± 0.01A	0.17 ± 0.00AB	0.15 ± 0.00B
	MAP2	0.21 ± 0.01A	0.21 ± 0.02A	0.19 ± 0.01A	0.15 ± 0.00Bb	0.14 ± 0.00Bb
	Vacuum	0.21 ± 0.01	0.21 ± 0.00	0.20 ± 0.00	0.19 ± 0.006a	0.18 ± 0.00a
C20:4 n-6	MAP1	5.44 ± 0.05	5.42 ± 0.07	5.34 ± 0.07	5.30 ± 0.09	5.23 ± 0.05a
	MAP2	5.44 ± 0.05A	5.35A ± 0.06A	5.19 ± 0.03A	5.04 ± 0.06B	4.79 ± 0.13bB
	Vacuum	5.44 ± 0.05	5.41 ± 0.05	5.39 ± 0.02	5.34 ± 0.06	5.32 ± 0.07a
C20:5 (EPA)	MAP1	0.57 ± 0.01A	0.55 ± 0.02A	0.53 ± 0.02A	0.5 ± 0.01Aa	0.43 ± 0.01Bc
	MAP2	0.57 ± 0.01A	0.57 ± 0.01A	0.55 ± 0.02A	0.42 ± 0.01Bb	0.33 ± 0.01Cb
	Vacuum	0.57 ± 0.01	0.56 ± 0.01	0.55 ± 0.01	0.52 ± 0.02a	0.51 ± 0.01a
C22:6 (DHA)	MAP1	0.67 ± 0.02A	0.64 ± 0.01A	0.62 ± 0.02A	0.59 ± 0.02B	0.45 ± 0.01Cb
	MAP2	0.67 ± 0.02A	0.63 ± 0.01A	0.61 ± 0.02A	0.53 ± 0.01Bb	0.25 ± 0.09Cc
	Vacuum	0.67 ± 0.02	0.67 ± 0.01	0.65 ± 0.01	0.63 ± 0.02a	0.65 ± 0.09a

Mean values bearing different letters either for each day within rows (A, B, C) or between packaging systems within columns (a, b, c) differ significantly at *p* < 0.05. ND, not detectable = [100 − ∑(SFA + MUFA + PUFA)].

**Table 5:** Sum of estimated FA (%) and FA ratio (mean ± SE) in ostrich meat as related to the type of packaging and refrigerated storage.

Fatty Acid (%)	Method	Day				
		0	4	8	12	16
SFA	MAP1	31.33 ± 0.21	31.42 ± 0.15	31.55 ± 0.14	31.62 ± 0.14	31.8 ± 0.16b
	MAP2	31.33 ± 0.21B	31.43 ± 0.11B	31.48 ± 0.18B	31.59 ± 0.18B	33.08 ± 0.09Aa
	Vacuum	31.33 ± 0.21	31.39 ± 0.19	31.46 ± 0.09	31.52 ± 0.19	31.58 ± 0.24b
MUFA	MAP1	38.56 ± 0.16	38.69 ± 0.16	38.11 ± 0.21	37.41 ± 0.13	36.91 ± 0.19
	MAP2	38.56 ± 0.16	38.68 ± 0.21	38.39 ± 0.20	37.88 ± 0.20	37.36 ± 0.17
	Vacuum	38.56 ± 0.16	38.57 ± 0.16	38.13 ± 0.16	37.66 ± 0.13	37.37 ± 0.12
PUFA	MAP1	28.48 ± 0.10A	28.30 ± 0.16A	27.70 ± 0.14AB	27.24 ± 0.18Ba	26.38 ± 0.16Cb
	MAP2	28.48 ± 0.10A	28.30 ± 0.17B	27.31 ± 0.13Cb	26.39 ± 0.16Db	24.69 ± 0.21Ec
	Vacuum	28.48 ± 0.10A	28.41 ± 0.14A	28.08 ± 0.07a	27.61 ± 0.13Ba	27.61 ± 0.13Ba
PUFA n-6	MAP1	24.21 ± 0.11A	24.1 ± 0.16A	23.63 ± 0.14AB	23.33 ± 0.18B	22.90 ± 0.12Ba
	MAP2	24.21 ± 0.11A	24.13 ± 0.19A	23.30 ± 0.13B	22.85 ± 0.19B	22.07 ± 0.20Cb
	Vacuum	24.21 ± 0.11A	24.15 ± 0.13A	23.90 ± 0.07AB	23.52 ± 0.12B	23.52 ± 0.12Ba
PUFA n-3	MAP1	3.21 ± 0.04A	3.16 ± 0.02A	3.09 ± 0.04A	2.98 ± 0.02Aa	2.72 ± 0.04Bb
	MAP2	3.21 ± 0.04A	3.14 ± 0.03A	3.05 ± 0.04A	2.69 ± 0.06bB	2.05 ± 0.03Cc
	Vacuum	3.21 ± 0.04	3.20 ± 0.03	3.15 ± 0.03	3.08 ± 0.04a	3.08 ± 0.04a
PUFA n-6/n-3	MAP1	7.55 ± 0.12	7.63 ± 0.07B	7.66 ± 0.11B	7.84 ± 0.10a	8.43 ± 0.09Ab
	MAP2	7.55 ± 0.12C	7.68 ± 0.10C	7.66 ± 0.13C	8.53 ± 0.23Bb	10.78 ± 0.13Aa
	Vacuum	7.55 ± 0.12	7.55 ± 0.07	7.60 ± 0.09	7.65 ± 0.11a	7.65 ± 0.11c

Mean values bearing different letters either for each day within rows (A, B, C) or between packaging systems within columns (a, b, c) differ significantly at *p* < 0.05. ND, not detectable = [100 − ∑(SFA + MUFA + PUFA)].
